# Investigation of Cross-Linked and Additive Containing Polymer Materials for Membranes with Improved Performance in Pervaporation and Gas Separation 

**DOI:** 10.3390/membranes2040727

**Published:** 2012-10-22

**Authors:** Katharina Hunger, Nadine Schmeling, Harold B. Tanh Jeazet, Christoph Janiak, Claudia Staudt, Karl Kleinermanns

**Affiliations:** 1 Institute of Physical Chemistry, Heinrich-Heine-University, Düsseldorf 40225, Germany; Email: katharina.hunger@hhu.de; 2 Institute of Organic Chemistry and Macromolecular Chemistry, Heinrich-Heine-University, Düsseldorf 40225, Germany; Email: nadine.schmeling@uni-duesseldorf.de; 3 Institute of Inorganic and Structural Chemistry, Heinrich-Heine-University, Düsseldorf 40225, Germany; Email: harold.tanh.jeazet@uni-duesseldorf.de; 4 BASF SE, Advanced Materials and Systems Research, Carl-Bosch Str., Ludwigshafen 67056, Germany

**Keywords:** pervaporation, separation, photocrosslinking, membranes, permeability, permselectivity, mixed-matrix membranes, mechanism, crosslinking degree, photochemistry, metal-organic frameworks, MOFs, porosity

## Abstract

Pervaporation and gas separation performances of polymer membranes can be improved by crosslinking or addition of metal-organic frameworks (MOFs). Crosslinked copolyimide membranes show higher plasticization resistance and no significant loss in selectivity compared to non-crosslinked membranes when exposed to mixtures of CO_2_/CH_4_ or toluene/cyclohexane. Covalently crosslinked membranes reveal better separation performances than ionically crosslinked systems. Covalent interlacing with 3-hydroxypropyldimethylmaleimide as photocrosslinker can be investigated *in situ* in solution as well as in films, using transient UV/Vis and FTIR spectroscopy. The photocrosslinking yield can be determined from the FTIR-spectra. It is restricted by the stiﬀness of the copolyimide backbone, which inhibits the photoreaction due to spatial separation of the crosslinker side chains. Mixed-matrix membranes (MMMs) with MOFs as additives (fillers) have increased permeabilities and often also selectivities compared to the pure polymer. Incorporation of MOFs into polysulfone and Matrimid^®^ polymers for MMMs gives defect-free membranes with performances similar to the best polymer membranes for gas mixtures, such as O_2_/N_2_ H_2_/CH_4_, CO_2_/CH_4_, H_2_/CO_2_, CH_4_/N_2_ and CO_2_/N_2_ (preferentially permeating gas is named first). The MOF porosity, its particle size and content in the MMM are factors to influence the permeability and the separation performance of the membranes.

## 1. Introduction

Membranes processes are very important in our every day life but also in industry, e.g., for water and waste water treatment, in medical applications or separation of petrochemicals. Classical separation methods used for purification of chemical products, notably distillation, extraction and crystallization are energy and cost intensive. Over 50% of the energy costs in the chemical industry are used for the separation of gaseous or liquid mixtures [1]. With membrane technology, the costs for difficult separations, e.g., of azeotropic mixtures, can be reduced significantly [2].

Membranes with pore sizes of more than 2 nm made out of ceramics, zeolithes, glass, metal or polymers are frequently used in practice [3,4,5]. Here, separation is based on size exclusion and thus these membranes are suited for separation of components with sufficient size difference, e.g., in dialysis, waste water treatment and functional clothing. For other applications, especially for separation of components with comparable sizes or the separation of ions from water, solution-diffusion membranes are used [6].

The solution diffusion mechanism is based on the principle that the mixture component with higher solubility and higher diffusion rate permeates preferentially through the membrane, independent on the component sizes [7,8]. Solution-diffusion membranes feature free volume sites which cannot be occupied by polymer chains due to restricted motion and packing density of the polymer chains. The components are transported through the membrane by successive movement between the transient free volume gaps close to the feed side to those close to the permeate side due to thermal motion of segments of the polymer chains [9].

**Figure 1:**
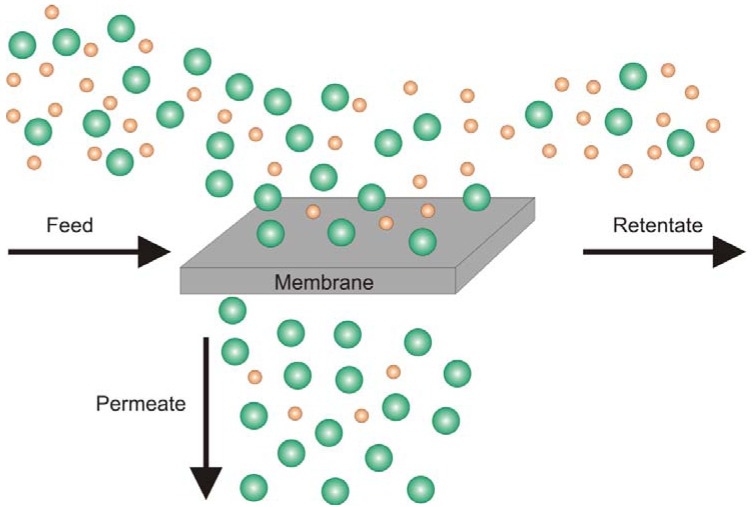
Principles of pervaporation. The liquid feed mixture ﬂows along the membrane and the feed components diffuse into and through the membrane at different rates. The liquid retentate is depleted and the vaporous permeate enriched in the preferentially permeating component [12].

The pervaporation process, which is used for the separation of liquid mixtures, is schematically shown in Figure 1. The liquid mixture (feed) whose components shall be separated is led over the membrane. Before permeation a phase transition from the liquid phase to the vapour phase is induced by thermal heating. The component which permeates preferentially is concentrated in the permeate; whereas the detained component is enriched in the retentate. Which component is preferentially permeating is dependent on the solubility of the components in the polymer matrix and the diffusion rate of the components through the membrane. The driving force for permeation is given by the difference of chemical potentials of the components on feed and permeate side respectively, depending on pressure, temperature and concentration difference [8,10,11]. In gas separation, the feed as well as retentate and permeate are gaseous and no phase transition occurs. 

The separation characteristics of a membrane are classified in terms of permeability *P* and selectivity *α*. The permeability is a measure of the permeate's quantity while the selectivity describes its quality (mole fraction of preferentially permeating component in the permeate) [9]. 

There are different types of materials which are suitable for membrane application. Inorganic membranes like ceramic membranes of metal oxides (γ-Al_2_O_3_, ZrO_2_, TiO_2_ or ZrTiO_4_), perovskites or zeolites, porous carbon membranes, metal membranes or porous glass membranes [13] have exceptionally high permeability and selectivity together with thermal and chemical stabilities. However, manufacturing procedures are more complicated (e.g., require support treatment, zeolite crystallization, thermal programming for pyrolysis and a controlled inert gas atmosphere in terms of flow, pressure and composition) and there is a lower reproducibility for membrane properties associated with high cost and low mechanical stability. This makes the production of inorganic membranes more difficult than for polymer membranes [13,14,15].

Therefore, most of the commercial solution-diffusion membrane units contain polymeric materials [16,17,18,19], which can be divided into rubbery and glassy polymers. While glassy polymers show very attractive separation characteristics, rubbery polymers show comparably low selectivity with high permeability for common gas pairs such as O_2_/N_2_, H_2_/CH_4_, CO_2_/CH_4_, *etc.* [20,21]. Of the glassy polymers, polyimides have been found to be very promising as membrane materials because they not only have better separation characteristics than the commonly used ones (e.g., polycarbonates) but also offer good thermal and chemical resistance and are easy to process [9]. Additionally they can be easily functionalized and therefore customized for any given separation problem. 

Solution-diffusion membranes already find application in removal of CO_2_ from natural gas, dehydration of organic solvents, desalination, removal of polar low molecular weight components in equilibrium reactions, oxygen or nitrogen enrichment from air as well as in vapour recovery systems and several more [22,23,24,25]. 

Of those applications, natural gas treatment is of the highest industrial interest [26]. Currently, less than 5% of the plants processing natural gas employ membrane technology, which is mainly used for removal of carbon dioxide [27]. As mentioned above, processing costs can be reduced significantly by substitution or extension of the commonly used amine absorption plants [28] by membrane separation processes. Natural gas is composed of mainly methane (75%–90%) and some higher hydrocarbons. Unfortunately, a significant number of natural gas resources cannot be exploited due to the high amount of CO_2_ (up to 30%) and other undesirable impurities (water, nitrogen, hydrogen sulfide, *etc.*) contained in the mixture, since they lead to corrosion of the pipelines [23]. The specifications for natural gas delivery to the U.S. national pipeline grid [29] demand carbon dioxide contents of less than 2%. First polymeric membranes for removal of CO_2_ from natural gas consisted of cellulose acetate [24]. Cellulose acetate membranes have a CO_2_/CH_4_ selectivity of about 12–15 under normal operating conditions, which is not sufficient for industrial application [23]. Today, more and more polyimides are used as membrane material instead of cellulose derivatives, since the separation properties are better (selectivities of 20–25) [9,23]. However, the low durability of unmodified polyimide membranes poses a problem, which can be overcome by crosslinking [9,30]. 

Besides natural gas treatment, separation of aromatics and aliphatics is a promising field for application of solution-diffusion membrane technology. In Europe, the benzene percentage in motor fuel is limited to 1 vol.% by law [31]. Reduction of the benzene content of reformates is usually done by extraction with usage of a tetraethylene glycol/water mixture as the extracting solvent. Here, the separation factor for the aromatics is between 2 and 3 [32]. By implementing pervaporation membranes, the cost-intensive extracting unit can be replaced. The pervaporation properties of many different polymers were studied in respect to an application as membrane material for aromatics/aliphatics separation, *i.e.*, polyimides, polyphosphonates, acetyl cellulose, benzoylated chitosan or polyurethane-silica hybrid membranes. The benzene/cyclohexane selectivity obtained with those polymers as membrane material typically lies between 5 and 20, with a normalized flux of 5–10 kg µm m^−2^ h^−1^ [33,34,35]. It is important that the membrane material is stable and that the separation characteristics will not change drastically in dependency on the concentration of aromatics in the reformate. Although all of those polymers show promising pervaporation characteristics, the stability often recedes at high aromatic concentrations and elevated feed temperatures. Again, crosslinking of the polymer can lead to an increased durability of the membrane [32]. As was shown for methyl methacrylate/methacrylic acid copolymer membranes ionically crosslinked with metal ions, the separation factor and normalized flux are not smaller than those of the non-crosslinked polymers [36].

The separation of olefin/paraffin mixtures is rather difficult because of the small differences in physical properties. Currently, such separations are carried out by low temperature distillation, which is rather cost intensive. Although membrane based hybrid processes would be less cost intensive they are not applied, since the separation factors of the membrane materials currently commercially available (e.g., silicone rubber, polysulfone, cellulose acetate, 1,2-polybutadiene or polyethylene) are too low [37,38]. Very attractive separation characteristics can be achieved with polyimides as membrane materials [39,40,41,42,43], but these materials are very sensitive to plasticization. To obtain membrane materials suitable for olefin/paraffin separation on industrial scales, the problem of plasticization has to be resolved, first. Some strategies for increase of membrane durability are presented in this review.

Gas separation membranes used in industrial applications are usually integrated as hollow fibers or flat sheets packaged as spiral-wound modules [27]. They are composed of a thin nonporous gas-selective layer (typically less than 0.5 µm [23]) deposited onto a highly porous carrier layer [8,11,44]. The carrier material increases the mechanical stability of the membrane, without influencing the separation characteristics [17]. A promising advance is the usage of composite membranes, in which different materials are used for the two different layers. This way, materials for both layers can be optimized separately and the high-cost polymer materials necessary for good separation properties can be applied more economically, since they are only needed for the very thin selective layer [27].

A membrane material is well suited for separation, when permeability and selectivity both are high. By modification of the polymer, these parameters can be increased. Unfortunately, improvement of the permeability often is gained in conjunction with a loss of selectivity, or the other way around [17,20,45]. For several polycarbonates, poly(ether sulfone)s and polyimides the solubility selectivities for O_2_/N_2_ and CO_2_/CH_4_ were found to be very similar, independent on the polymer structure, while the selectivities varied significantly [11,46]. That implies, that diffusion selectivity *D_x_/D_y_* is of higher importance in gas separation than solubility selectivity *S_x_/S_y_*, according to Equation (1) [17].


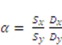
(1)

Therefore, to optimize both permeability and selectivity, a narrower free volume distribution in combination with a higher chain stiffness should be attempted [47]. 

**Figure 2:**
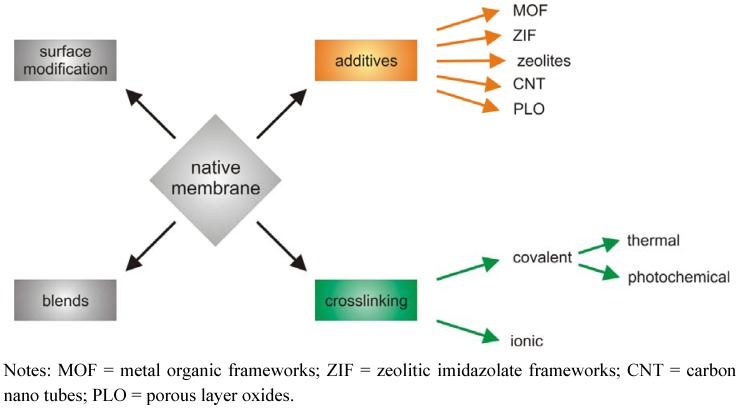
Different approaches to improve polymeric membrane materials.

The most common approaches for membrane modification are shown in Figure 2. Usually at least two polymers are blended together, to take advantage of the different properties of those components. For example, it was found that monomers having bulky substituents, e.g., the 6FDA dianhydride which contains -CF_3_ groups (see Figure 8 for structure), restrict chain mobility and simultaneously chain packing, and lead to significantly improved selectivity as well as permeability [46,48]. Today, most copolymers consist of alternating bulky and flexible monomer units are used and by now a variety of monomers is available [17]. 

Although permeability and selectivity are the main criteria for classification of the separation characteristics of a membrane, other properties of the material have to be considered as well, as already mentioned above. The membranes are required to be thin but nonetheless stable, and they are desired to be low in cost. Additionally to mechanical stability, the chemical resistance against the feed mixture is of great importance. 

Membrane fouling, that is adsorption of impurities on the membrane surface or into the membrane, can lead to significant decreases in permeability of the membrane. By surface modification, e.g., grafting adequate monomers on the surface or plasma treatment, the surface roughness, hydrophilicity and charge can be adjusted to the particular feed mixture and thus prevent or confine fouling [49].

Native, *i.e.*, unmodified, polymeric membranes often show strong plasticization effects when exposed to high partial pressures of CO_2_ [50,51], hydrocarbons [52,53] or ethylene oxide [54]. This leads to an increase in intermolecular distance and molecular motion of the polymer chains due to a decrease of inter- and/or intramolecular attractive forces. Consequently, the permeability for all feed components increases whereas the selectivity decreases. Strong plasticization can even lead to a partial dissolution of the membrane [32]. The hydrocarbons present in natural gas can reduce the selectivity by 30%–50% [50,55]. The effect is larger for rubbery polymers than for glassy polymers, which implies that plasticization is a problem especially for polyimides. This can be confined by implementing either covalent or ionic crosslinks of the polymer backbone. In both cases the plasticization resistance [30,53,54,56] as well as the separation efficiency can be improved [57,58,59,60] compared to the non-crosslinked polymer for pervaporation [36,61] and gas separation [62,63,64,65].

**Figure 3:**
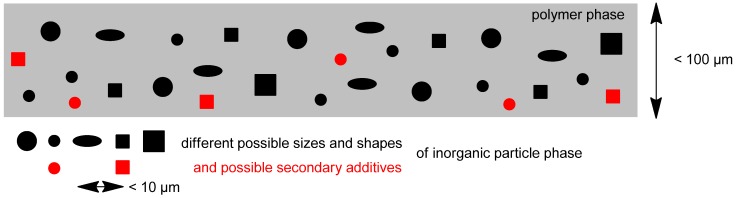
Schematic representation of a mixed-matrix membrane indicating the different possible sizes, shapes and components for the inorganic filler materials (e.g., MOFs, ZIFs and nanotubes).

Another approach to increase the separation characteristics of a membrane is the implementation of additives. Mixed-matrix membranes (MMMs) are constructed from an inorganic material in the form of discrete micro- or nanoparticles incorporated into a polymeric matrix as the continuous phase (see Figure 3). The utilization of two materials with different flux and selectivity opens the possibility to improve a gas separation membrane by allowing the synergistic combination of polymers with their easy processability and the superior gas separation performance of inorganic materials. 

A strong improvement of permeability could be expected, if porous inorganic additives, e.g., zeolites, metal organic frameworks (MOFs) [66,67,68,69,70,71,72], carbon nanotubes (CNTs) [73,74,75,76,77,78,79,80,81] or porous layered oxides (PLOs) [82,83,84,85,86,87,88] with a pore diameter larger than the kinetic diameter of one gas component but smaller than the other one could be applied to the organic polymer. 

**Figure 4:**
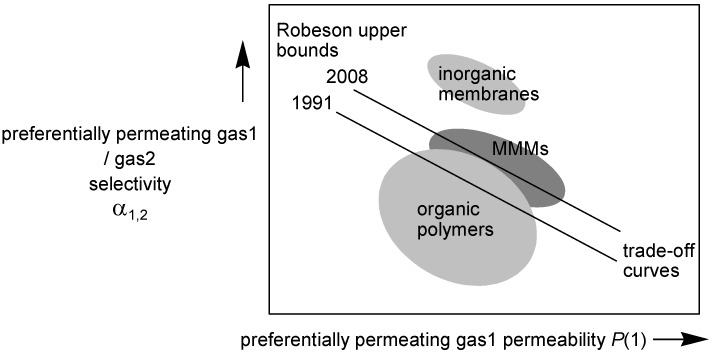
Schematic presentation of the trade-off between permeability and selectivity with the 1991 and 2008 Robeson upper bounds [20,21]. In most cases the technologically attractive region lies around or above the Robeson upper bound.

This would be economically very attractive for large scale applications if the selectivity does not change. First MMMs were studied in the 1980s and are more and more investigated in recent years [89,90,91,92,93]. MMMs receive attention as a possibility to enhance the properties of pure polymer membranes [94]. Separation properties with MMMs can be well above the Robeson upper bond (see Figure 4) [20,21], which is a plot of permeability *versus* selectivity for most industrial relevant gas mixtures. Porous inorganic fillers can overcome the tradeoff between selectivity and permeability which is typical for continuous (pure) polymer membranes.

Carbon nanotubes (CNTs) have been potentially suggested for their application in molecular separation by considering their hollow channels. In fact, simulations and detailed predictions demonstrated that the diffusivities of light gases inside the pores of CNTs are highly rapid compared to other porous materials [73]. According to this potential application, the use of CNT/polymer nanocomposites in membrane based separation processes is growing rapidly and considerable efforts were undertaken for the development in last decade [74,75,76,77,78,79,80] CNT particles are added as fillers to create preferential permeation pathways and thereby improve the separation performance in terms of the gas permeability [76,81]. The processability of polymeric membranes is combined with the higher permeability of nanotubes in order to achieve a synergistic separation performance that surpasses that of conventional polymeric and inorganic membranes.

To increase the gas permeability and also enhance the selectivity behavior of polymer membranes, addition of nanoporous fillers have been intensively investigated [90,93,95,96,97,98]. However, zeolites particle face the problem of formation of aggregates creating defects, especially if the polymer matrix is consisting of a glassy polymer [99]. Several attempts of modifying the surface of the zeolites to chemically bond them to the polymer chains and avoid macrovoid formation have been made, which often led to a considerable decrease of the gas permeability due to the rigidification of the polymer chains near the zeolite surface [100,101]. Also modification of the zeolite surface by using amino functionalized siloxan to induce the formation of hydrogen bonds has been used [102]. 

Furthermore, interest has risen in sheet-shaped porous layer oxides (PLOs) such as TiO_2_ [89], SiO_2_ [103,104,105,106] and Na_4_Ti_2_Si_8_O_22_·4H_2_O [87,88] as fillers which are also called flakes or nanosheets if one of the dimensions is in the nanometer range. The aforementioned materials in lamellar form have been used as inorganic fillers in mixed-matrix membranes [87,88]. Such sheet-shaped or lamellar fillers have a high aspect ratio, which allows casting of thin mixed-matrix membranes. The selectivity of the membrane can be improved by size exclusion due to their microporous structure. Such filler materials are subdivided into selective and non-selective fillers depending on the permeability of the feed components through these materials. Also, such sheet-shaped fillers can be incorporated into MMMs in random or distinct parallel orientation. It was previously shown that the orientation of non-permeable inorganic sheets in different polymers has a great influence on the permeability [82,83]. Similar improvement of the separation characteristics can be achieved with smaller amounts of oriented fillers as with higher loads of non-oriented filler. Predictions of the permeation properties of mixed-matrix membranes with sheet-shaped inorganic fillers like porous layered oxides have been made for oriented non-selective sheets [84] as well for selective flakes [85]. Another approach to oriented sheet membranes dealt with fully inorganic MCM-22/silica nanocomposite membranes [86].

## 2. Crosslinking

Crosslinking of polymer membranes can be achieved by different approaches, depending on the functional groups of the polymer and the desired separation properties of the membrane. The aim is to increase the durability of the membrane by circumvention of plasticization without causing losses in selectivity and permeability. Ideally, the separation characteristics of the membrane, especially the selectivity, are enhanced by implementation of crosslinks. 

Often there is a variety of approaches to induce crosslinking of a polymer. For instance, a detailed review of different crosslinking methods for poly(vinyl alcohol) (PVA) membranes is provided by Bolto *et al*. [107]. PVA membranes show high stability in highly acidic or alkaline environments [108]. They are often chosen for dehydration procedures, where swelling due to adsorption of water presents a problem. 

By performing several freeze-thaw cycles, crystalline regions are formed that act as crosslinks [109]. With increasing crystallinity the swelling is reduced. Although the physical crosslinks are not as strong or as stable as chemical ones, membranes modified this way last several months without a change of separation properties [107,110]. Reduction of plasticization of PVA can also be achieved by heat treatment at temperatures up to 160°, but the permeability is decreased as well. This, too, is explained by a change in crystallinity rather than the introduction of covalent crosslinks [111,112].

Treatment of PVA with strongly oxidizing radical producers (e.g., K_2_S_2_O_8_) leads to formation of polymer radicals, which form crosslinks by radical coupling. This has also been shown to reduce the solubility of the polymer [113]. By varying the amount of persulfate the crosslinking degree can be regulated [114].

### 2.1. Ionic Crosslinking

**Figure 5:**
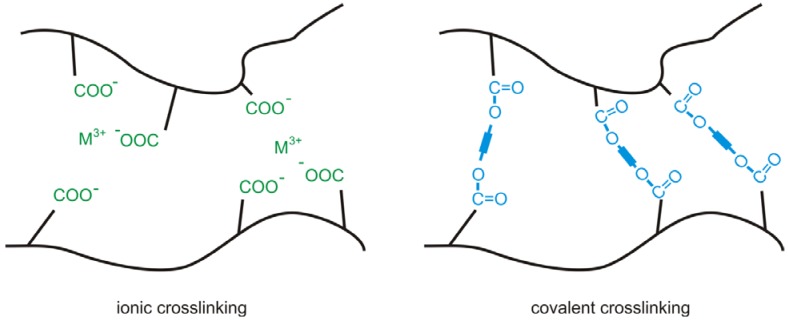
Possible crosslinking methods in polymers containing carboxyl groups. In ionic crosslinked polymers the metal ions form a complex with several deprotonated carboxyl groups. Covalent crosslinking can be achieved thermally or photochemically.

Crosslinking of copolyimides can be achieved by different ways, since they can be easily functionalized and thus modified [115]. Copolyimides containing carboxyl groups can be ionically crosslinked by thermal reaction of the dissolved polymer with a stoichiometric amount of aluminium(III)-acetylacetonate or zircon(IV)-acetylacetonate (Figure 5). The metal ions form a complex with three (Al^3+^) or four (Zr^4+^) deprotonated carboxyl groups in a heterogeneous distribution of regions with ionic and non-ionic domains. Therefore the membrane exhibits polymer regions which are sensitive to plasticization in the presence of strong plasticizers such as CO_2_ [9,30]. The acetylacetonate evaporates during the crosslinking reaction, but not necessarily to completeness. The remaining anions can cause problems in separation due to elution which leads to an undesirable change of the membrane performance and contamination of the retentate. In addition, the strong solvation by CO_2_ is able to weaken the ionic interactions in the ionic regions. 

### 2.2. Thermal Crosslinking

In order to separate mixtures which contain high amounts of strong plasticizers, the membrane is better crosslinked covalently [30,115] (Figure 5). Covalent crosslinking can be achieved by thermal reaction of the dissolved polymer with the crosslinking agent (e.g., diols or CNTs [116]). It was shown that covalent binding of fullerenes to poly(2,6-dimethyl-1,4-phenylene oxide) leads to improved separation characteristics for gas separation in respect to the native membrane, while implementation of dispersed fullerenes leads to worse separation characteristics, due to formation of fullerene agglomerates [117]. 

Other studies, using dispersed CNTs instead of fullerenes, present more promising results [118,119]. However, the long term stability of those membranes has not been investigated and it is assumed that a CNT-loss due to membrane swelling occurs. This can be prevented by binding the CNTs covalently to the polymer. Copolyimides crosslinked with CNTs are for example well suited for the removal of sulfur containing aromatics from fuel. Sorption capacity tests showed that the covalent incorporation of hydroxyl-functionalized CNTs does not affect the solution selectivity of the copolyimide [88], while the mechanical stability of the membranes is enhanced [120].

Non-crosslinked PVA membranes contain hydroxyl groups, which can form hydrogen bonds with water. By crosslinking of PVA with formaldehyde, the chemical stability of the membrane material can be significantly increased but the amount of free hydroxyl groups is reduced, which results in reduced sorption rate of water vapour in the PVA membrane [121]. However, crosslinking with formaldehyde demands application of sulphuric acid catalysts, which is assumed to result in negatively charged membranes [122,123]. 

PVA membranes are also used in reverse osmosis. It was found that upon crosslinking with different dicarboxylic acids with increasing number of carbon atoms in the crosslinker the water flux increased, while the salt rejection was decreased, due to the increased flexibility of the crosslinks [124]. Covalent crosslinking of PVA with maleic anhydride leads to lower water fluxes but higher separation factors than found for non-crosslinked PVA in the dehydration of benzene [121]. In another study, PVA crosslinked with maleic anhydride showed not only an higher separation factor (dehydration of 2-propanol and 1-butanol) but also a higher water flux [125]. Although the same crosslinker was used in both studies, the effect of crosslinking on the separation characteristics is different. This leads to the conclusion that mechanistic studies of crosslinking reactions are of great importance, since they provide vital information for the control of polymer crosslinking. 

For these reactions, catalysts are necessary, which remain in the membrane after the crosslinking reaction and thus can lead to the same disadvantages as the anions in ionic crosslinking. One strategy to obtain covalent crosslinks without the usage of catalysts was presented by Evonik. Here, the polyimide is formed by polycondensation of a tetracarboxylic acid anhydride and an aromatic diisocyanate and then treated with a diamine solution. The crosslinking reaction takes place at moderate temperatures (preferentially 20–50 °C) without addition of further reactants [126].

### 2.3. Photocrosslinking

Another promising strategy to circumvent these problems is to achieve the covalent crosslinking by a photochemical reaction, since in this case no catalyst is needed [127,128,129,130,131,132]. The photochemical reaction has to be efficient without usage of sensitizers, since sensitizers would again cause the problems in separation mentioned before. First approaches based on direct crosslinking of the polymer backbone had to deal with very large permeability losses due to the increased stiffness of the polymer network and decreased free volume of the polymer [33,133,134]. By functionalisation of polymer carboxyl groups with flexible molecules (e.g., ethylene glycol or propanediol), this disadvantage can be overcome [50,135,136,137]. Depending on stiffness and length of the crosslinker, the permeability loss of the membrane can be minimized [138]. Thus, functionalization of the polymer carboxyl groups with flexible molecules capable of dimerization upon UV irradiation (e.g., maleimide derivatives) is a promising strategy to obtain membranes with a high plasticization resistance and excellent separation characteristics [127,139,140]. A detailed summary of polymer materials and photo-crosslinkers suitable for gas separation and pervaporation was recently provided by He *et al.* [141]. 

Photocrosslinking of a polyimide with benzophenone as crosslinking unit was performed by Kang *et al.* [142]. They found that the O_2_ permeability decreased and the O_2_ over N_2_ selectivity increased with increasing irradiation time. The same results were obtained for H_2_/CH_4_ separation [63]. Here, the selectivity increased by the factor of 50 after 30 min of irradiation, with a decrease in H_2_ permeability by the factor of 5. With increasing irradiation time, the larger crosslinking degrees were obtained, which caused further increases in gas permselectivity and decreases in gas permeability. Thus, by varying the irradiation time, the separation characteristics can be tuned. 

Since most of the UV light was absorbed by the benzophenone, the irradiation intensity became smaller with increasing penetration depth. Thus an inhomogeneous distribution of crosslinks was obtained [65,143]. This results in lower crosslinking degrees than achievable with thermal activation and thus improvements in separation selectivity with only modest reduction in fast gas flux are obtained. In contrast, thermal activation of the same system results in larger improvements in chemical resistance, thermal stability and gas selectivity but also a more significant reduction of gas flux, as was shown for a copolyimide with crosslinkable ethynyl-terminated monomers [144].

Benzophenone forms radicals upon irradiation, which may lead to undesirable side reactions [145]. Thus molecules like maleimide, which are capable of forming crosslinks via [2+2]-cycloaddition present an interesting alternative for application in gas separation or pervaporation membranes [127,139,140]. Unfortunately, up to now, no detailed study of the separation characteristics of membranes crosslinked with maleimide is available.

#### 2.3.1. Mechanistic Studies of Photocrosslinking

**Figure 6:**
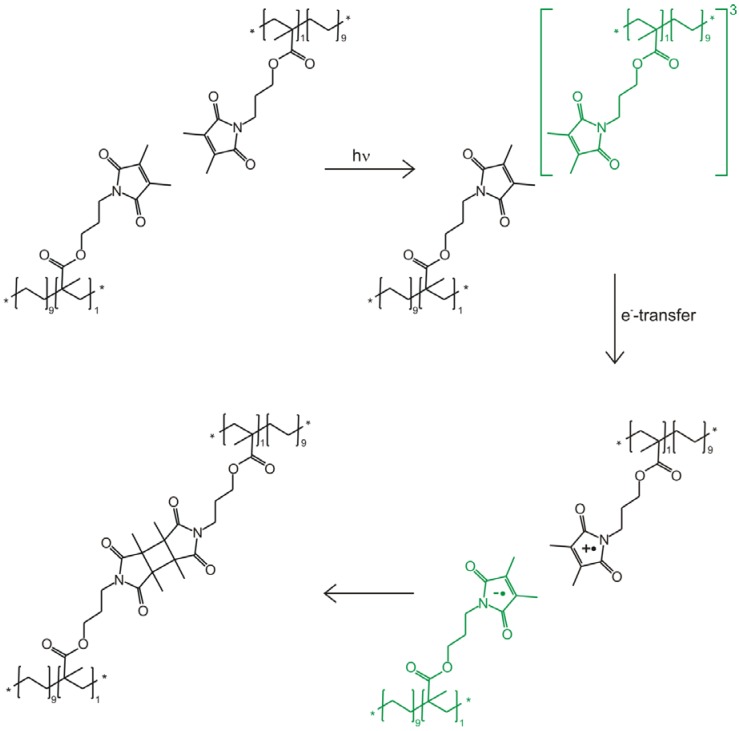
Mechanism for photocrosslinking of PEMAA modiﬁed with 3-hydroxypropyldimethylmaleimide. Upon excitation to the bright singlet state some population is transferred to the triplet state by intersystem crossing. Electron transfer leads to quenching of the triplet population and allows spectroscopic identification of the maleimide anion. Photocrosslinking occurs by recombination of the ions to the dimer. Our spectroscopic results do not exclude a parallel reaction path via direct cross linking of favorably oriented maleimides in the excited singlet state, see text. Transient species which were detected spectroscopically (triplet state, radical anion) are marked in green.

Mechanistic studies of photocrosslinking reactions can be performed using UV/Vis absorption spectroscopy. This was done for polymer membranes containing maleimide moieties as crosslinker [146]. Maleimides undergo dimerization by [2+2]-photocycloaddition (see Figure 6) [127,147,148]. Due to the high photoactivity of the maleimides no sensitizers are needed for this reaction. Thiomaleimides absorb better and at longer wavelengths than maleimide and thus perform photodimerization with increased efficiency [149]. However, the separation properties of membranes which are crosslinked with thiomaleimides remain to be further investigated. 

Copolyimides feature intense absorption in the same spectral region (210–250 nm) as maleimide. Therefore, the reaction mechanism is better studied with a polymer backbone which does not absorb UV-light. Here, poly[ethene-stat-(methacrylic acid)] (PEMAA) represents a good alternative, because the separation properties of crosslinked PEMMA membranes are comparable to those of glassy copolyimides having carboxylic acid groups. In either case, crosslinking of the polymer material results in decreased swelling without significant loss of selectivity [58]. 

It is known that crosslinking of a polymer influences the glass transition temperature, T_g_ [150]. The glass transition temperature depends highly on the polymer chain mobility. Crosslinkers which enlarge the packing density of the polymer limit the mobility of the polymer chains, which results in higher T_g_. Crosslinkers which function as spacer between the polymer chains or have a high flexibility increase the polymer chain mobility and thus induce a decrease of T_g_ [151]. PEMAA with 6% MI shows a higher glass transition temperature (58 °C) compared to the non-crosslinked copolymer (48 °C) [146]. Thus, by functionalization of the PEMAA with maleimide the polymer chain mobility is reduced. Analogous observations have also been made for covalently crosslinked glassy copolyimides, like 6FDA-4MPD/6FDA-DABA 4:1 (see Figure 8 for structure) [152]. 

The stationary UV/Vis absorption spectrum of PEMAA films esterified with 3-hydroxypropyl-dimethylmaleimide (MI) shows an absorption band at 230 nm just as maleimide solutions do [153]. This absorption disappears completely after 20 min of irradiation with a mercury lamp and does not recover after the irradiation has been stopped, which indicates that photochemical crosslinking occurs efficiently and irreversible [146].

**Figure 7:**
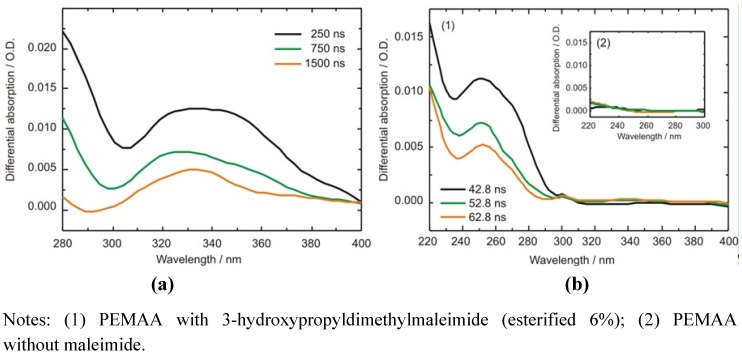
Transient absorption spectra obtained at different time delays to the excitation laser pulse of (**a**) PEMAA esterified with 3-hydroxypropyl-dimethylmaleimide (esterification degree of 6%) in THF upon excitation at 266 nm. The broad absorption at ~340 nm is due to triplet-triplet excitation in maleimide [154]; (**b**) PEMAA films upon excitation at 266 nm. Absorption between 264 nm and 268 nm is dominated by scattered light and therefore the measured absorption in this range is omitted from the spectral average (five data points: ~8 nm).

Microsecond transient UV/Vis absorption investigations of maleimide modified PEMAA in tetrahydrofuran (THF) provide evidence that, after excitation with a 266 nm laser pulse, intersystem crossing takes place and the triplet state is populated (see Figure 7a). The broad absorption band at around 340 nm is effectively quenched by oxygen. The decay time of the triplet state in solution was determined to be 2.58 ± 0.23 µs, see also Reference [146].

In films prepared from the same solution no triplet absorption is observed anymore. Nonetheless, nanosecond transient absorption spectra of the maleimide modified PEMAA films feature an absorption band with a maximum at 250 nm (see Figure 7b). Films which do not contain the maleimide side group show no absorption in this spectral region. This band was observed in pulse radiolysis experiments as well and was assigned to the maleimide radical anion [154,155]. The maleimide radical anion lifetime in the PEMAA film is determined to be 18.05 ± 0.36 ns. Our time resolution, limited to ~15 ns, does not allow exclusion of a parallel singlet reaction path via direct (concerted) cross linking of favorably oriented maleimides in the excited singlet state. In the film, crosslinking is not quenched by oxygen which indicates that dimerization occurs faster than oxygen diffusion. 

In the PEMAA membranes described above only 6% of all carboxylic acid groups were esterified with MI. Upon irradiation the maleimide absorption band vanishes completely, which means that nearly all maleimide groups have formed crosslinks. At higher esterification degrees PEMAA becomes insoluble and production of films is not possible. 

**Figure 8:**
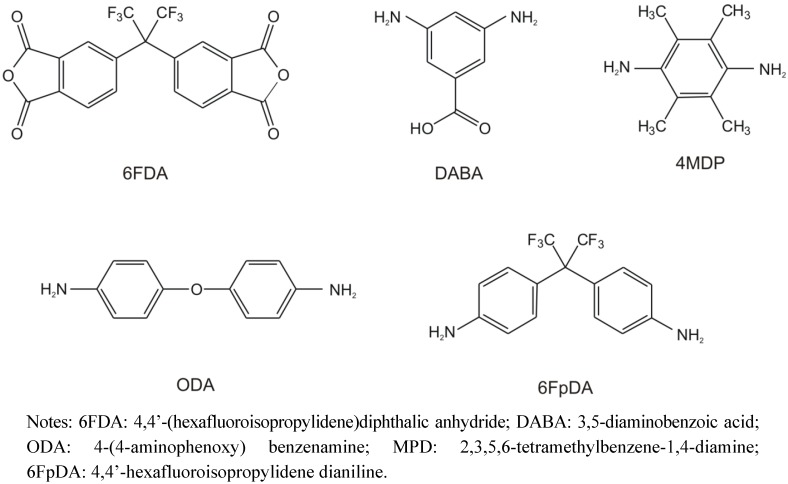
Chemical structures of the copolyimid components discussed in this review.

As mentioned before, UV/Vis absorption spectroscopy is not suitable to investigate photocrosslinking of copolyimide membranes with maleimide as crosslinking unit. However, this can be achieved by FTIR difference spectroscopy, a method by which only bands arising from vibrations which change during the monitored reaction appear in the spectrum. Band positions of negative peaks represent the unshifted positions of the disappearing educts; positive bands can be assigned to those vibrations of the photoproducts, which exhibit different frequencies than the educts. The strong absorption of the polymer backbone is not subject to changes caused by the photoreaction [156] and is thus not visible in the FTIR difference spectra. This method is non-invasively and can be performed in real time which is a great advantage over other established methods as the determination of gel fractions [157], weight ratios [158], swelling properties [159] or rheology [160,161]. Additionally, structural information about the membrane and the photoproducts can be obtained.

**Figure 9:**
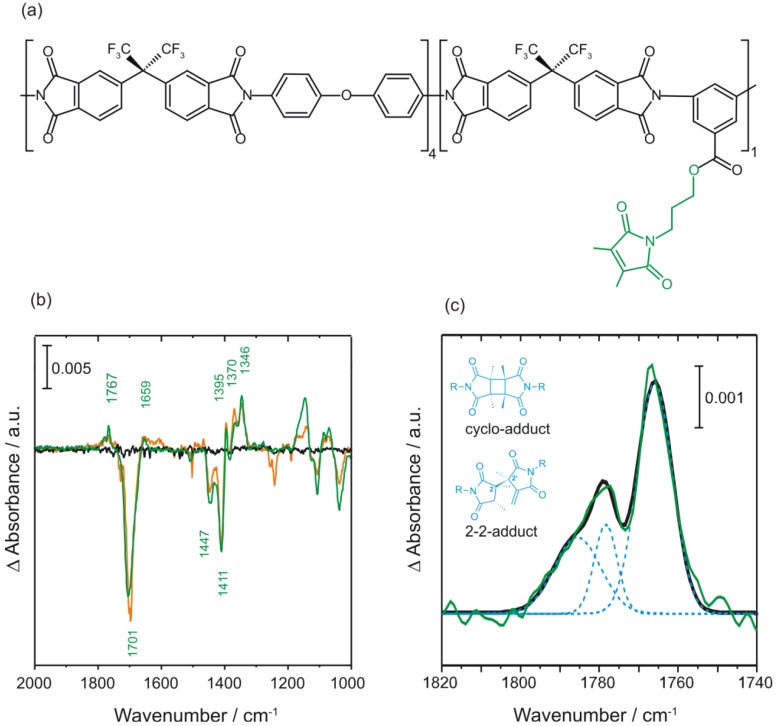
(**a**) Chemical structure of 6FDA-ODA/6FDA-DABA 4:1 copolyimide functionalized with 3-hydroxypropyldimetylmaleimide. Depicted in green is the maleimide side group; (**b**) Laser-induced FTIR difference spectra of 3-hydroxypropyldimethyl-maleimide (green), 6FDA-ODA/6FDA-DABA 4:1 copolyimide (black) and the copolyimide with 3-hydroxypropyldimethylmaleimide (green); (**c**) Difference spectrum of 3-hydroxypropyldimethylmaleimide (green) in the carbonyl region. The positive feature can be reproduced by the sum of three Gauss curves (blue).

An extensive FTIR difference absorption study was performed on 6FDA-ODA/6FDA-DABA 4:1 films functionalized with MI. The structures of the different monomes used for the synthesis of the copolyimides investigated are shown in Figure 8. The structure of the 6FDA-ODA/6FDA-DABA 4:1 polymer is shown in Figure 9a. Although it was intended to obtain complete functionalization, that is each DABA moiety carries a maleimide moiety (theoretical functionalization degree: 100%), it cannot be excluded, that free carboxyl groups remain in the polymer.

Comparison between films of (i) 6FDA-ODA/6FDA-DABA 4:1, (ii) 6FDA-ODA/6FDA-DABA 4:1 with MI and (iii) MI shows, that appreciable photochemistry only occurs in the presence of the crosslinker (see Figure 9b). The difference spectrum of the copolyimide without maleimide moiety does not exhibit any light-induced difference bands. The difference spectra of MI containing films feature a positive band in the region of 1800–1750 cm^−1^ which is shown in detail in Figure 9c. It derives from a superposition of three bands which cannot be generated by the main photoproduct, the *trans-*cycloadduct, alone. According to DFT calculations the *cis*-cycloadduct (see Figure 9c for the structure) cannot be distinguished easily by infrared spectroscopy from the *trans-*cycloadduct, since there are no prominent marker bands. However, due to its drilled ring system, an additional carbonyl vibration shows significant IR activity and thus contributes to the absorption spectrum [156]. 

Formation of a third photoproduct, the 2-2'-product (see Figure 9c for the structure), is assumed due to the appearance of a positive absorption band at 1659 cm^−1^, which corresponds to the terminal C=C double bond stretching vibration. This photoproduct was also detected in solutions of different N-alkyl-3,4-dimethylmaleimides [162] and polyacrylamide hydrogels functionalized with dimethyl-maleimide [160]. No evidence was found for the formation of an oxetane-like photoproduct, which would result from a Paterno-Büchi-reaction [163].

The time constants for photoproduct accumulation upon irradiation with laser pulses with 266 nm and 6 mJ cm^−2^ are in the range of several minutes. Prominent bands assigned to the cycloadduct appear with τ = 2 to 5 min while the 2-2'-adduct marker band increases with τ = 75 min. This indicates that the 2-2'-adduct is formed with a lower yield than the cycloadduct. Both adduct species absorb at 266 nm better than the monomeric maleimide [162], therefore it is not unlikely that the 2-2'-adduct is not only formed directly from the maleimide monomers but also by rearrangement of the cycloadduct. Photodegradation of the polymer backbone competes with photocrosslinking. Biexponential fits of the negative difference absorption bandswith time constants τ_1_ = 5 min and τ_2_ > 120 min were obtained. The slower process is assigned to photodamage of the polymer backbone and the faster process to photocrosslinking.

#### 2.3.2. Photocrosslinking Yield

The separation characteristics of a crosslinked membrane are strongly influenced by the number of crosslinks in the membrane. This value is of greater significance for pervaporation membranes than the amount of polymer chains which were crosslinked [157,160]. When using a photocrosslinker, the crosslinking yield can be controlled by duration and intensity of the irradiation. 

The yield can be determined with the help of FTIR difference absorption spectroscopy, by calculating the ratio of difference absorbance ∆A to initial absorption A. Since multiple processes compete and many absorption bands are generated by superposition of product and reactant absorption, determination of the crosslinking yield by analyzing a single prominent band may not be sufficient [127,128,159,160]. An analysis of several bands, after Gauss deconvolution to correct for the spectral overlap, is more likely to deliver significant results.

For a 6FDA-ODA/6FDA-DABA 4:1 copolymer with MI it is assumed that only maleimide side chains that react within τ_1_ = 5 min contribute to product formation. In order to avoid falsification due to overlapping product bands, the quantitative analysis is restricted to the region between 1450 cm^−1^ and 1400 cm^−1^ where products do not absorb significantly. After subtraction of the contribution due to photodegradation, the difference absorbances of the negative bands at υ_1_ = 1447 cm^−1^ and υ_2_ = 1411 cm^−1^ are calculated to be ∆A_1_ = 0.010 and ∆A_2_ = 0.018. The initial absorptions A_i_, derived from the absorbance spectrum of the copolyimide with MI, are A_1_ = 0.16 and A_2_ = 0.28. With these values, a crosslinking yield ∆A/A of 6.4% is calculated for both bands.

For polyurethane-acrylate crosslinked with maleimide, crosslinking yields of 50% are reported [164], while with polymethacrylate as polymer backbone crosslinking yields are even higher (up to 90%) [127]. These high values are obtained when the polymer backbone is of high flexibility. The stiffness of the 6FDA-ODA/6FDA-DABA 4:1 polymer backbone prevents close vicinity of the excited maleimide moieties to a second maleimide during the lifetime of the radical anion as necessary for crosslinking. This effect is higher in films than in solution. The low crosslinking yield is not necessary a disadvantage. High crosslinking yields lead to low flow rates and can cause the membrane to be brittle, which makes the material inapplicable for separation. Thus, it is desirable to fabricate membranes with a crosslinking yield high enough to prevent plasticization but not too high so that the obtained flow rates are still high enough to qualify for industrial applications.

### 2.4. Pervaporation and Gas Separation studies of Crosslinked Membranes

Copolyimides show excellent separation characteristics for different gaseous and liquid mixtures. Here, aromatic/aliphatic separation and the removal of carbon dioxide from natural gas with high CO_2_ content are discussed in detail. The crosslinking yield of the PEMAA membranes with MI discussed above was too low to prevent plasticization efficiently. The membranes of 6FDA-ODA/6FDA-DABA 4:1 with MI were very brittle and thus not suited for pervaporation experiments. However, sorption capacity measurements of the photocrosslinked 6FDA-ODA/6FDA-DABA 4:1 membrane show decreased swelling degrees and increased sorption selectivities in respect to the native membrane material [165]. The best results for covalently crosslinked membranes in pervaporation performance studies were obtained with 6FDA-4MPD/6FDA-DABA 4:1 crosslinked by thermal reaction of the polymer backbone with 1,4-butanediol [165]. 

#### 2.4.1. Aromatic/Aliphatic Separation

It has been shown that crosslinked membranes can be conditioned, that means treated with high aromatic feed mixtures prior to use. This leads to a more open structure and results in a higher flux also in long term experiments without a significant loss in selectivity, whereas non-crosslinked copolymides swell very strongly or even partially dissolve at high aromatic concentrations [32]. 

For comparison of covalently, ionically and non-crosslinked 6FDA-4MPD/6FDA-DABA 4:1 membranes, pervaporation experiments using a toluene/cyclohexane mixture were performed. The ionically crosslinked membrane was prepared from the basic polymer material by addition of zircon(IV)-acetylacetonate (theoretical functionalization degree: 10%). The covalently crosslinked membrane was prepared from the basic polymer material by addition of 1,4-butanediol in a six-fold excess over the amount of carboxylic acid groups and with toluenesulfonic acid as catalyst at 150 °C (theoretical functionalization degree: 100%).

**Figure 10:**
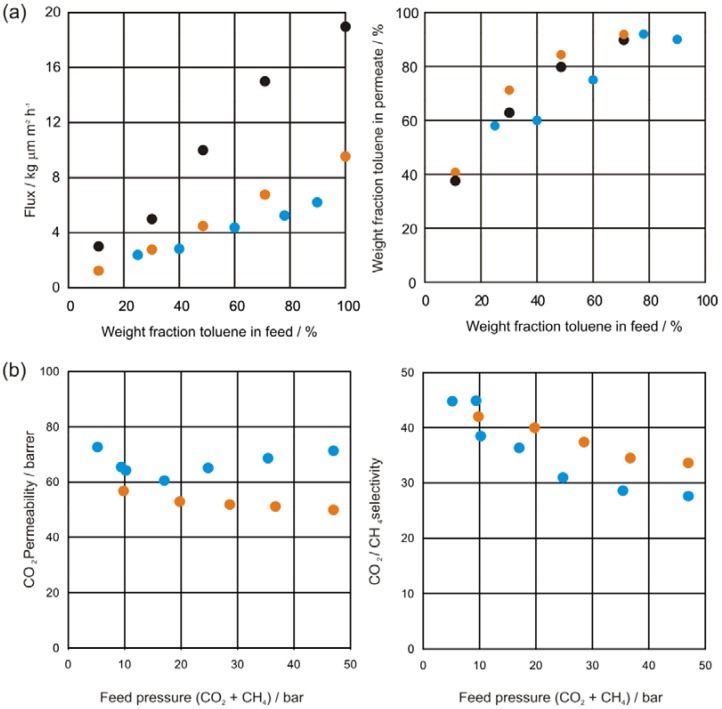
(**a**) Pervaporation results for conditioned 6FDA-4MPD/6FDA-DABA 4:1 copolyimide membranes, crosslinked with 1,4-butanediol (orange) and crosslinked with zircon(IV)-acetylacetonate (blue) and non-crosslinked (black) using a toluene/cyclohexane mixture at 60 °C. Permeate pressure was kept between 20 and 25 bar [9]; (**b**) CO_2_/CH_4_ separation characteristics for the 6FDA-6FpDA/6FDA-4MPD/6FDA-DABA 3:1:1 copolyimide ionically crosslinked with aluminium(III)-acetylacetonate (blue) and covalently crosslinked with ethylene glycol (orange) at 35 °C using a 50:50 CO_2_/CH_4_ feed gas mixture [9].

The results obtained show that the selectivity is slightly higher for covalently crosslinked membranes with respect to the non-crosslinked ones and lower for ionically crosslinked membranes (see Figure 10a). The flux is significantly lowered by covalent crosslinking and even more so by ionic crosslinking. This effect increases with increasing aromatic feed concentrations. Whereas usually the feed concentration for the aromatics in the reformate stream ranges between 40% and 50%, much higher concentrations occur occasionally [9]. 

It is obvious that in both cases of crosslinking, ionically as well as covalently, the flux is reduced compared to the non crosslinked membrane material. However the strong increase of flux with increasing aromatic content in feed is suggesting swelling of the membrane. Comparing the performance of the crosslinked membrane materials it can be seen from Figure 10a that the fluxes of both membrane types do not vary significantly. However, although the selectivity of ionically and covalently crosslinked membranes are comparable at high-range aromatic concentrations, covalently crosslinked membranes should be favored for this kind of separation since a better long term performance is expected.

#### 2.4.2. Natural Gas Treatment

Plasticization is also a problem for separation of CO_2_ from natural gas. For non-crosslinked copolyimides it generally occurs at 10–20 bar partial CO_2_ pressure in the feed. Crosslinked copolyimides not only offer plasticization resistance up to much higher CO_2_ pressures but also have a better chemical resistance.

The non-crosslinked copolyimide 6FDA-4MPD and ionically crosslinked 6FDA-4MPD/6FDA-DABA 4:1 start plasticizing at a CO_2_ pressure of approximately 15 bar whereas the covalently crosslinked 6FDA-4MPD/6FDA-DABA 4:1 membrane is resistant to plasticization up to CO_2_ pressures of more than 30 bar [9]. These results are in good agreement with those found for other copolyimides [30]. With both crosslinked membranes the permeability is decreased whereas the selectivity is not changed significantly compared to the non-crosslinked systems. 

By substitution of 75% of the 4MPD by 6FpDA the selectivity can be increased up to commercially attractive values. 6FpDA contains bulky -CF_3_ groups which cause restricted rotation around the main polymer chain and also provide a higher free volume. The permeability of the covalently crosslinked 6FDA-6FpDA/6FDA-4MPD/6FDA-DABA 3:1:1 membranes is nearly constant for CO_2_ partial pressures of 5 to 50 bar, whereas the ionically crosslinked membranes plasticize approximately at 20 bar (Figure 10b). With covalent crosslinking, the selectivity is approximately 20% higher than with ionic crosslinking. In both cases, a decrease in selectivity was found with increasing feed pressure.

Most importantly, the crosslinked membranes show good CO_2_/CH_4_ selectivity even above CO_2_ partial pressures of 40 bar, and thus qualify for commercial applications since the separation factors are much higher than those of the cellulose derivatives in current use [166].

## 3. Metal-organic Frameworks in Mixed Matrix Membranes

Metal-organic frameworks (MOFs) are of increasing interest as porous filler in mixed-matrix membranes (see Figure 3). MOFs offer various advantages over zeolites or other porous inorganic additives [167,168,169,170,171]: The organic ligands as an inherent part of MOFs allow them to interact well with the polymer material and its functionalities. This way, the formation of gaps between the “inorganic” filler and the organic polymer phase, which would cause losses in selectivity, can be avoided. Also the MOF surface properties can be easily tuned by functionalisation with various organic molecules if necessary. Moreover, MOFs also have higher pore volumes and lower density than zeolites, and therefore their effect on the membrane properties can be more pronounced for a given mass loading. For MMMs a perfect interaction between the two components is highly important in order to achieve optimized separation properties of the hybrid material.

**Figure 11:**
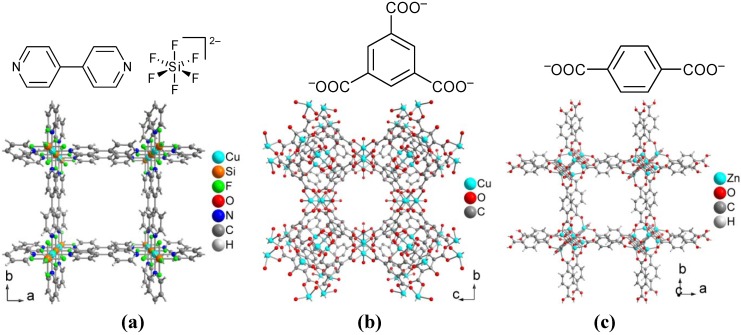
Linkers and sections of the packing diagrams of (**a**) Cu-BPY-HFS, [Cu(µ-SiF_6_)(µ-4,4'-bipy)_2_] (BPY = 4,4'-bipyridine, HFS = hexafluorosilicate), (**b**) Cu-BTC**, **[Cu_3_(BTC)_2_(H_2_O)_3_] (BTC = benzene-1,3,5-tricarboxylate) and (**c**) MOF-5 (IRMOF-1), [Zn_4_O(BDC)_3_] (BDC = benzene-1,4-dicarboxylate, terephthalate).

Incorporation of the MOF 4,4'-bipyridine-hexafluorosilicate-copper(II) (Cu-BPY-HFS) (see Figure 11a) into a dense Matrimid^®^ 5218 membrane increased the gas permeabilities and selectivities for pure gases (from single gas experiments) and gas mixtures of CH_4_/N_2_ but decreased the ideal CO_2_/CH_4_ and H_2_/CH_4_ selectivities [172].

Cu-BTC MOF (see Figure 11b) as additive in an asymmetric membrane of Matrimid^®^ 9725 or of 3:1 Matrimid^®^/polysulfone Utrason S 6010 N blend showed a higher CO_2_ permeance compared to the unfilled membrane in mixed gas permeation experiments [173,174]. The binary gas mixtures CO_2_/CH_4_ and CO_2_/N_2_ were studied for CO_2_ concentrations from 10 vol.% to 75 vol.%. The permeance of the preferential permeating gas CO_2_ and, hence, the CO_2_ selectivity increased with the filler loading. Noteworthy, the CO_2_/CH_4_ selectivity α_CO_2_/CH_4__ dropped linearly when the CO_2_ content increased from 10 vol.% to 75 vol.%. The slope of this selectivity drop did not depend on the [Cu_3_(BTC)_2_] additive content [173,174]. This could be due to plasticization phenomena with higher CO_2_.

Cu-BTC with polyimide (PI) prepared from 4,4-oxydianiline (ODA) and pyromellitic dianhydride (PMDA) was successfully spun into MMM hollow fibers by a dry/wet-spinning method. H_2_ permeance and selectivity of H_2_ with respect to N_2_, CO_2_, O_2_ and CH_4_ increased with increased Cu-BTC loading. At 6 wt.% Cu-BTC, the permeance of H_2_ was higher by 45%, and its ideal selectivity from other gases was up by a factor of 2–3 compared to pure PI [175].

**Figure 12:**
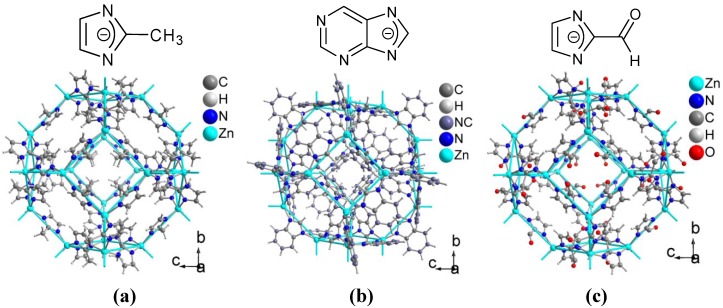
Linkers and sections of the packing diagrams emphasizing the cuboctahedral b-cage which is depicted by blue topological lines connecting the Zn atoms of (**a**) ZIF-8, [Zn(2-methylimidazolate)_2_], (**b**) ZIF-20, [Zn(purinate)_2_] and (**c**) ZIF-90, [Zn(2-carboxyaldehyde imidazolate)_2_].

Cu-BTC (see Figure 11b) or ZIF-8 MOF (see Figure 12a) and the zeolite silicalite-1 (S1C) were combined in a polysulfone Udel^®^ P-3500 MMM [176] for the separation of CO_2_/N_2_, CO_2_/CH_4_, O_2_/N_2_ and H_2_/CH_4_ mixtures. For some of these gas mixtures, the combined-filler MMM showed a synergetic enhancement in selective gas transport when compared either to the pure polymer or to the MMM with only one filler type. All fillers, at the same loading of 16 wt.%, increase the CO_2_ permeability when compared to that of the bare polymer in the two CO_2_-containing mixtures. However, the maximum separation selectivities for CO_2_/CH_4_ and CO_2_/N_2_ mixtures (α_CO__2__/CH_4__= 22.4 with *P* = 8.9 barrer for CO_2_, and α_CO__2__/N2_= 38.0 with *P* = 8.4 barrer for CO_2_) were only achieved when Cu-BTC was combined with S1C in a PSF MMM [176].

When the same Cu-BTC/ZIF-8/S1C single- or double-additive MMMs were applied to O_2_/N_2_ and H_2_/CH_4_ separations, for which mechanism is based mainly on diffusion and not on adsorption differences, the combination of Cu-BTC and S1C significantly enhanced the selectivities of O_2_ or H_2_, respectively. This may be due to an improved diffusion through zeolite crystals from the synergy with Cu-BTC [176].

MOF-5 as nanocrystals showed a strong affinity to the polymer matrix of Matrimid^®^ 5218. In MOF-5, six carboxylate groups coordinate to the six edges of the tetrahedral {Zn_4_O} unit in octahedral geometry and the terephthalate linker then form the edges of a cube in the primitive cubic network (see Figure 11c). Single gas permeabilities of H_2_, CO_2_, O_2_, N_2_ and CH_4_ doubled approximately with MOF-5 loadings of 30 wt.% from those of the pure polymer. This increase was ascribed to the MOF-5 porosity. Tests with binary CH_4_-containing gas mixtures for the MMM with 30% MOF-5 in the polyimide revealed an increase in selectivity for CH_4_. The larger solubility of CO_2_ and N_2_ in the polymer matrix was seen as the reason to favor enhanced CH_4_ transport [177].

Zeolitic imidazolate framework-8 (ZIF-8,) with Matrimid^®^ 5218 MMMs was prepared with loadings up to 80% (w/w), which are much higher than the typical loadings achieved with selected zeolite materials [178]. In ZIF-8, the 2-methylimidazolate ligands connect individual zinc atoms and form the edges of a cuboctahedral -cage in a sodalite network (see Figure 12a). The ZIF-8/Matrimid^®^ MMMs permeabilities were tested for H_2_, CO_2_, O_2_, N_2_, CH_4_, C_3_H_8_, and gas mixtures of H_2_/CO_2_ and CO_2_/CH_4_ and increased as the ZIF-8 loading increased to 40% (w/w). This increase in permeability suggested that the addition of ZIF-8 nanoparticles to Matrimid^®^ increases the distance between polymer chains creating higher free volume in the polymer matrix. For the majority of gas pairs (O_2_/N_2_, CH_4_/N_2_, H_2_/O_2_, H_2_/CO_2_, H_2_/N_2_), there was no significant change in the ideal selectivity until 40 wt.% ZIF-8 loading. However, at higher loadings of 50 wt.% and 60 wt.%, the permeability decreased for all gases, and the selectivities increased. Ideal selectivities of gas pairs containing gases of different size, such as H_2_/O_2_, H_2_/CO_2_, H_2_/CH_4_, CO_2_/CH_4_, CO_2_/C_3_H_8_ and H_2_/C_3_H_8_, showed improvement with the 50 wt.% ZIF-8 loading through a shift from polymer-driven to ZIF-8-controlled gas transport process. ZIF-8 can selectively transport smaller gas molecules, such as H_2_ and CO_2_ [178].

An asymmetric MMM of Matrimid^®^ 9725 and ZIF-8 (or MIL-53(Al), see below) exhibited a higher CO_2_ permeance than the unfilled membrane in mixed gas permeation experiments [174]. MOF fillers with 10 wt.%, 20 wt.% and 30 wt.% were loaded in Matrimid^®^. Binary gas mixtures CO_2_/CH_4_ and CO_2_/N_2_ with CO_2_ concentrations from 10 vol.% to 75 vol.% were investigated and the results compared to Cu-BTC/Matrimid^®^ [173] or MIL-53(Al) [174]. The permeance of the preferentially permeating gas CO_2_ increased with the filler loading in the binary mixtures in all three cases but the CO_2_ selectivity only increased slightly in the case of Cu-BTC or MIL-53(Al) and remained almost constant for ZIF-8. The CO_2_/CH_4_ selectivity α_CO_2_/CH_4__ showed a constant drop when the CO_2_ content increased from 10 vol.% to 75 vol.% for all three additives. The selectivity drop was independent of the filler content. Increase in CO_2_ permeance was assigned to the extra pore network of the MOF fillers. Scanning electron microscopy cross-section images showed the MOF filler well distributed and embedded in the polymer matrix even for the 30 wt.% loaded membranes [173,174].

MMMs made of poly-(1,4-phenylene ether-ether-sulfone) (PPEES) and ZIF-8 with filler loadings of 10 wt.%, 20 wt.% and 30 wt.% were employed in CO_2_ diffusion studies using pulsed field gradient (PFG) NMR techniques [179]. The self-diffusion coefficient increased from 2.1 × 10^−8^ cm^2 ^s^−1^ for the pristine PPEES membrane to 9.3 × 10^−8^ cm^2 ^s^−1^ for the 30 wt.% ZIF-8/PPEES-MMM. ZIF-8 provides Langmuir adsorption sites for CO_2_ molecules, thus, the gas adsorption in the MMM increases with the filler content. ZIF-8 contributes greatly to gas permeation by increasing the gas solubility in the composite membranes [179].

Combining both ZIF-8 and silicalite-1 in polysulfone Udel^®^ P-3500 (ZIF-8/S1C-PSF MMM) did not improve the separation results from either S1C-PSF or ZIF-8-PSF MMMs in the case of CO_2_/CH_4_ and CO_2_/N_2_ gas mixtures. Probably the relatively large silicalite-1 crystals could not be intercalated between small ZIF-8 particles. ZIF-8 alone produced the highest increase of CO_2_ permeability, which can be attributed to its textural properties and its small particle size, albeit giving poorly dispersed aggregates [176]. For O_2_/N_2_ and H_2_/CH_4_ gas separation a ZIF-8-PSF MMM produced the best selectivity-permeability results compared to a Cu-BTC- or S1C-PSF MMM. This may be due to an increase in free volume (as suggested for ZIF-8-polyimide MMMs) [178] together with an efficient molecular separation effect (based on diffusion differences) because of the small pore aperture window of ZIF-8 (3.4 Å, 0.34 nm) compared to Cu-BTC (6 Å, 0.6 nm) and S1C (5.5 Å, 0.55 nm) [176].

Different sonication powers were applied on ZIF-8 nanoparticles for the preparation of ZIF-8/Matrimid^®^ nanocomposite membranes with the aim of investigating the effect of typical membrane processing conditions on the structure, the interfacial morphology and the gas separation performance. It was shown that ultrasonication generates significant changes in the shape, size distribution, and structure of ZIF-8 particles suspended in an organic solvent during membrane processing. Although there are significant changes in the particle morphology, there are only minor losses in crystallinity and microporosity as proven from powder X-ray diffraction, synchrotron X-ray pair distribution function analysis and nitrogen physisorption. Dynamic light scattering and electron microscopy show that ZIF-8 nanoparticles undergo substantial Ostwald ripening when subjected to high intensity ultrasonication. Composite films prepared with both direct (high-intensity) and indirect (low-intensity) sonication show good adhesion between the polymer and ZIF-8 phases. However, films prepared using indirect sonication exhibit drastic agglomeration of nanoparticles while direct sonication produced ripened nanoparticles with variable dispersion. The ripened particles give lower pore volumes and lower surface areas compared to the as-synthesized material. ZIF-8/Matrimid^®^ composite membranes prepared from the two different sonication methods show significant differences in microstructure. Permeation measurements in membranes fabricated with high-intensity sonication show strong enhancement in permeability of CO_2_ and increased CO_2_/CH_4_ selectivity, in agreement with the Maxwell model. In contrast, composite membranes prepared with low-intensity sonication are found to be defective [180].

Small and less agglomerated ZIF-20 with 8 wt.% in a polysulfone Udel^®^ P-3500 mixed matrix membrane gave a better separation of an equimolar O_2_/N_2_ mixture than the pure polymer. As in ZIF-8 the imidazolate moiety in the purinate ligands connects individual zinc atoms and forms the edges of a cuboctahedral -cage but in a zeolite-A network (see Figure 12b). The increase in O_2_/N_2_ selectivity from 4.7 (±0.4) to 6.7 (±0.5) in the MMM could be justified by the small difference in kinetic size between O_2_ (kinetic diameter d_k_ = 0.343 nm) and N_2_ (d_k_ = 0.368 nm) [181].

Submicrometer-sized particles of ZIF-90 (see Figure 12b, sodalite network again as in ZIF-8) were used to fabricate nanocomposite membranes with three different polyimides [Ultem^®^ 1000, Matrimid^®^ 5218 and 6FDA-DAM (6FDA: 2,2-bis(3,4-carboxyphenyl)hexafluoro-propane dianhydride, DAM: diaminomesitylene)] [171]. Scanning electron microscopy revealed an excellent adhesion of ZIF-90 crystals with the polyimides with no interfacial voids and well dispersed MOF crystals. Ultem^®^ and Matrimid^®^ MMMs showed significantly enhanced CO_2_ permeability without any loss of CO_2_/CH_4_ selectivity. ZIF-90 with the highly permeable polymer 6FDA-DAM showed significant enhancements in both CO_2_ permeability and CO_2_/CH_4_ selectivity. Membranes containing smaller particles showed slightly better results. The performance of ZIF-90/6FDA-DAM MMM exceeded the polymer upper bound for polymeric membrane performance from 1991 [20], and reaches the technologically attractive region (see Figure 4) [171].

The CO_2_/CH_4_ binary mixture gas-permeation properties of pure 6FDA-DAM and 15 wt.% ZIF-90/6FDA-DAM membranes revealed an enhanced gas-separation performance of the MMM. The CO_2_/CH_4_ mixed-gas selectivity of the ZIF-90 MMM was higher than the ideal selectivity measured by single-component gas permeation, presumably because of selective sorption and diffusion of CO_2_ in the ZIF-90 crystals [171]. Also, a ZIF-90/6FDA-DAM membrane showed an ideal CO_2_/N_2_ selectivity of 22 compared to 14 for pure 6FDA-DAM indicating the possibility for separation of CO_2_ from flue gases [171].

**Figure 13:**
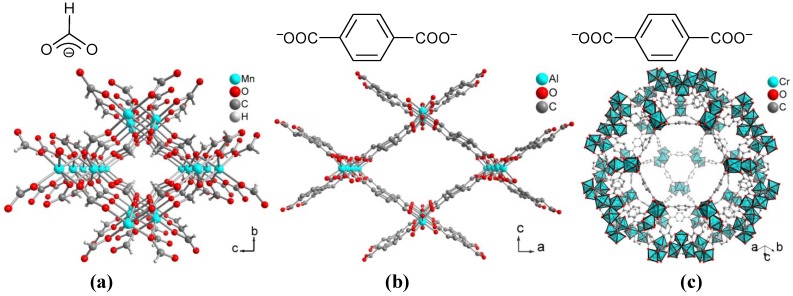
Linkers and sections of the packing diagrams of (**a**) Mn(HCOO)_2_, (**b**) MIL-53(Al), [Al(BDC)(µ-OH)] (BDC = benzene-1,4-dicarboxylate, tere-phthalate) and (**c**) MIL-101, [Cr_3_(O)(BDC)_3_(F,OH)(H_2_O)_2_].

The MOF Mn(HCOO)_2_, where each formiate ligand bridges between three manganese atoms (see Figure 13a), has very small channels of ~2 Å diameter only, and hence, as PSF-MMM showed high adsorption affinity for H_2_ only. Higher loadings reduced the gas solubility, but increased the permeability, indicating defective membranes with interfacial voids [182].

An asymmetric membrane from MIL-53(Al) (or Cu-BTC or ZIF-8, see above) and Matrimid® 9725 showed a higher CO_2_ permeance than the unfilled membrane in mixed gas permeation experiments [174]. The framework of MIL-53(Al) is a flexible, 'breathing'-type network, that is, it can assume different shapes and porosities depending on guest presence or absence. The BDC ligand bridges between four Al atoms. The hydroxo-bridging takes place along the metal chains in the b direction (see Figure 13b). The permeance of the preferentially permeating CO_2_ in the binary gas mixtures CO_2_/CH_4_ and CO_2_/N_2_ with CO_2_ concentrations from 10 vol.% to 75 vol.% increased with the filler loading for all three MOFs but the CO_2_ selectivity slightly increased only in the case of Cu-BTC or MIL-53(Al) and remained almost constant for ZIF-8. Under the same conditions the CO_2_ permeability remained invariant with the type of MOF for the CO_2_/N_2_ gas mixture. The CO_2_/CH_4_ selectivity α_CO_2_/CH_4__dropped linearly when the CO_2_ content increased from 10 to 75 vol.% for all three MOFs. The decrease in selectivity was essentially independent of the MOF filler content [173,174]. 

A variant of MIL-53(Al), namely compound NH_2_-MIL-53(Al) with the 2-aminobenzene-1,4-dicarboxylate linker (NH_2_-BDC) but the same structure as MIL-53(Al) was used to fabricate nanocomposite membranes with PSF Udel^®^ P-3500. The homogeneously distributed NH_2_-MIL-53(Al) particles showed excellent adhesion with the polysulfone without any additional compatibilization. 

In contrast to most reported membranes, CO_2_/CH_4_ separation selectivity of NH_2_-MIL-53(Al)/PSF-MMM increased with pressure due to the flexibility of the NH_2_-MIL-53(Al) filler [183].

Mixed-matrix membranes with the water-stable MOF MIL-101 in polysulfone Ultrason S 6100 N exhibited a remarkable four-fold increase (compared to pure PSF) in the permeability of O_2_ to technically needed values above 6 barrer and a simultaneous high selectivity for O_2_ over N_2_ of 5–6. The largest cage in this network with MTN zeolite topology is shown here with an inner diameter of ~34 Å and pore aperture windows up to ~16 Å. The benzene-1,4-dicarboxylate ligands bridge between trinuclear {Cr_3_O} building units (see Figure 13c). The MIL-101 particles showed very good adhesion with polysulfone and long term stability [184].

**Figure 14:**
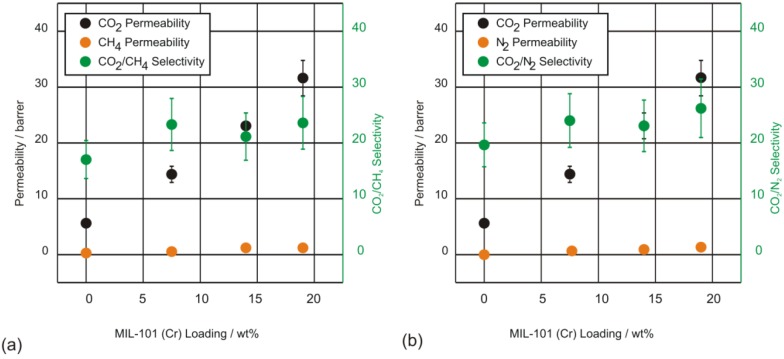
Single-gas CO_2_, CH_4_ and N_2_ permeabilities and ideal (**a**) CO_2_/CH_4_ and (**b**) CO_2_/N_2_ selectivities of pure PSF and MIL-101/PSF membranes with different MIL wt.% loadings (averaged values for different membrane thicknesses).

Single gas experiments of MIL-101/PSF membranes with CO_2_, CH_4_ and N_2_ at different MOF loadings showed increases in gas permeabilities with increasing MIL-101 weight percentage in PSF (see Figure 14). CO_2_ is the preferentially permeating gas with permeability increases from about 5 to over 35 barrer from pure PSF to 20 wt.% MIL-101/PSF. The increase for CO_2_ also raises the ideal selectivities for CO_2_/CH_4_ and CO_2_/N_2_ from about 20 to 25.

**Figure 15:**
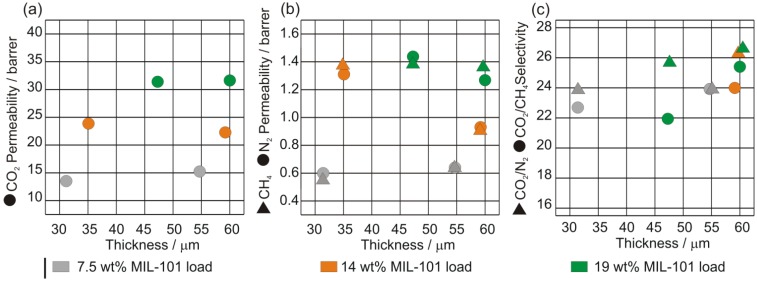
(**a**) Single-gas CO_2_; (**b**) CH_4_ and N_2_ permeabilities; (**c**) ideal CO_2_/CH_4_ and CO_2_/N_2_ selectivities for MIL-101/PSF membranes at different MIL wt.% loadings and membrane thicknesses.

The CO_2_ permeability is rather invariant to the thickness of the membrane while the CH_4_ and N_2_ permeabilities decrease when the membrane becomes thicker for the same MIL-101 loading (see Figure 15). Hence, the ideal CO_2_/CH_4_ and CO_2_/N_2_ selectivities strongly increase with the membrane thickness, in particular for CO_2_/CH_4_ (see Figure 15c).

## 4. Conclusions

Covalent crosslinking with maleimide derivatives as crosslinking unit can be investigated *in situ* in the liquid phase as well as in the solid phase using nanosecond transient UV/Vis absorption and FTIR measurements. Results point to maleimide anion formation via the triplet state and ionic dimerization to the cycloadduct as main reaction path upon UV irradiation. Additionally, the 2-2’-adduct is formed either from the educt or in a consecutive reaction from the cycloadduct. For 6FDA-ODA/6FDA-DABA 4:1 with MI the photocrosslinking yield was determined to be ~6%. The yield is restricted by the stiﬀness of the polymer backbone, which inhibits the photoreaction due to permanent spatial separation of the crosslinker side chains. 

It has been shown for the separation of high-pressure mixtures of CO_2_/CH_4_ as well as for toluene/cyclohexane mixtures that the photocrosslinked copolyimide membranes are much more plasticization resistant than noncrosslinked copolyimide membranes. Generally covalent crosslinking results in much higher plasticization resistance and better selectivity than ionic crosslinking.

Mixed matrix membranes with metal-organic frameworks as additives (fillers) exhibit enhanced permeabilities and possibly also selectivities when compared to the underlying pure polymer. Matrimid^®^ and polysulfone are popular polymer matrices for MOF fillers. MOF particle adhesion to these organic polymers does not represent a problem. Permeability increases can be traced to MOF porosity. Crucial permeability-selectivity factors worth further investigations are, *inter alia*, filler content, filler (nano)size and dispersion, gas-binding functional groups in filler (e.g., amines for CO_2_), water-stability of MOF-filler [185,186,187,188] as well as mixed-gases with different volume fractions instead of single gases. Addition of MOFs to polymers in MMMs easily yields performances similar to the best polymeric membranes and selectivities 10 times higher than those reported to date for any pure MOF membrane for the respective separation. In the end MOF-polymer MMMs allow for an easier synthesis and handability compared to pure MOF membranes [189,190,191,192,193].
